# A case of acute paraplegia that improved with dialysis

**DOI:** 10.4103/0972-5229.40950

**Published:** 2008

**Authors:** Govarthanan Rajendiran, Rajamahesh Jayabalan, Saravanan Chandrahasan, Ashwin Kumar Mani

**Affiliations:** **From:** Intensive Care Unit, Apollo First Med Hospitals, Chennai, India

**Keywords:** Acute paraplegia, hemodialysis, hyperkalemia

## Abstract

Acute severe hyperkalemia can present as acute paraplegia independent of cardiac effects, even though cardiac muscle is more sensitive to serum potassium changes. We managed a patient with acute hyperkalemic paralysis who did not have threatening cardiac/electrocardiographic manifestations. The limb weakness became normal after hemodialysis.

## Introduction

Acute severe hyperkalemia can present with arrhythmias, heart block, hypotension, sudden death, ascending flaccid paralysis and type II respiratory failure. Hyperkalemia commonly produces cardiac manifestations with typical electrocardiographic (ECG) changes because cardiac muscle is more sensitive to hyperkalemia. Classical ECG changes of hyperkalemia are: peaked T-waves, PR interval prolongation, flattening of P-wave, widening of QRS complex and “sine-wave” appearance at severely elevated levels.[1,2] Rarely acute severe hyperkalemia can present with acute paraplegia without life-threatening cardiac or typical ECG manifestations which requires early identification and immediate hemodialysis especially when serum potassium is lethally elevated or resistant to conservative measures.

## Case Report

A 60-year-old gentleman was admitted with the presenting complaint of acute onset weakness of both the lower limbs (started as inability to stand up after squatting), inability to move both the lower limbs and breathlessness. He had no other presenting complaints including history of back pain, trauma, chest pain, palpitations or syncope. He had bilateral cystic bronchiectasis of the lungs of unknown etiology, cor pulmonale and he was a diabetic on oral hypoglycemic agents (glibenclamide, glimepiride) and a hypertensive on atenolol and spironolactone. His other medications were frusemide and theophylline + etofylline.

On admission, he was conscious oriented and his vitals were: Temperature - 99°F, pulse-110/min, BP-130/80 mm Hg, RR-26/min reg and O_2_ saturation-95% with 10litres/min of oxygen flow. He had clubbing, bilateral pitting pedal edema and bilateral symmetrical areflexic motor weakness of the lower limbs (Power 1/5) and absent bilateral plantar response. Lung examination showed bilateral coarse crepitations. All peripheral pulses were equally felt and the rest of the system examination was unremarkable. His average urine output was > 1000 ml/day. His previous renal function tests were normal, with a normal serum potassium level. Magnetic resonance imaging (MRI) spine was advised by the neurologist.

Arterial blood gas analysis showed pH 7.28, pCO_2_ 60, pO_2_ 159 and bicarbonate 27.8. ECG showed normal sinus rhythm, right axis deviation, PR interval of 0.20 ms, right bundle branch block (RBBB), right ventricular hypertrophy (RVH) and ST depression/T inversion in leads III, aVF and V1-V3.

Laboratory investigations revealed blood sugar of 493 mg/dL, blood urea nitrogen (BUN) of 88 mg/dL, serum creatinine of 1.6 mg/dL, serum sodium of 119 mEq/L, serum potassium of 8.9 mEq/L, Hb-19.4g/dL, hematocrit-67% and WBC-8,200/cu.mm, creatine phosphokinase, was 95 IU L and thyroid profile was normal. Daily measurements of hemoglobin and PCV level did not show any evidence of ongoing hemolysis.

Chest X-ray and computed tomography chest showed bilateral cystic bronchiectasis of the lungs. Echocardiography showed dilated right atrium and ventricle, severe pulmonary artery hypertension, normal left ventricle size and systolic function and EF of 68%. Arterial Doppler of both lower limbs revealed thin plaques along the arteries with normal flow velocity.

Immediate treatment for hyperkalemia included intravenous calcium gluconate, nebulised salbutamol, insulin infusion and potassium binding resin and potassium free diet. Repeat serum potassium after one hour of conservative management was still 8.7 mg/dL. The Nephrologist started him on immediate hemodialysis, as the serum potassium did not respond adequately to medical therapy and as he was still at high risk of life-threatening cardiac events. Salbutamol nebulization and insulin infusion were continued. After one cycle of hemodialysis, medications and potassium restriction, his serum potassium was 7.1 mg/dL but his motor power improved to 4/5 in both the lower limbs (he was able to lift both his legs against gravity and mild resistance). Repeat ECG post-hemodialysis was the same. Imaging was not performed initially as he required urgent treatment for the hyperkalemia. Subsequently there was remarkable improvement in motor power, MRI spine was withheld.

He was kept on non-invasive ventilation which was discontinued the next day. He required three cycles of hemodialysis before his serum potassium reached the normal range. Throughout the course of the illness, he had no episode of arrhythmia, heart block or cardiac arrest. At discharge, his serum potassium and renal function were within the normal range. He was able to walk normally without any disturbances in posture or balance and his motor power was 5/5 in all the 4 limbs.

Follow-up was done after two weeks of discharge when he had normal motor power (able to walk and ride motorcycle) and normal potassium and renal function tests.

## Discussion

Acute hyperkalemic paraplegia in this patient was probably caused by a combination of factors including prerenal azotemia (due to right heart failure), beta-blocker, aldosterone antagonist and insulin deficiency. He required hemodialysis to bring the potassium level to a normal range and the rapid improvement of neurological symptoms was impressive. We report this interesting case of acute paraplegia caused by severe hyperkalemia without any typical ECG manifestations. There are three similar case reports of hyperkalemic paralysis without classic ECG changes.[3–5]

Szerlip *et al*. reported two cases of severe hyperkalemia (Serum potassium > 9.0 mEq/L) in whom the ECGs did not reveal the expected manifestations of hyperkalemia.[6] Shakil *et al*. suggested that the absence of ECG changes in hyperkalaemic hemodialysis patients should be interpreted with caution.[7] Wrenn *et al*. reported that ECG is not sensitive for detecting hyperkalemia even in high risk patients and empiric treatment of hyperkalemia based on ECG alone will lead to mistreatment of at least 15% of patients.[8] Martinez *et al*., reported seven hyperkalemic patients (K^+^ ≥ 8 mmol/L) without typical ECG changes.[9] So ECG is neither sensitive nor specific for diagnosis or to guide therapy of hyperkalemia.

Hyperkalemia should always be considered in the differential diagnosis of acute onset paraplegia even in the absence of typical ECG manifestations or cardiac effects. Hence ECG cannot be reliably used to exclude the presence of even potentially lethal serum potassium elevations or to monitor therapy of hyperkalemia.[6–9]
